# A Study of Physical, Chemical, and Sensory Characteristics of Novel Legume Dips

**DOI:** 10.1155/2024/2875348

**Published:** 2024-03-18

**Authors:** Lynda Makhloufi, Mohammad I. Yamani

**Affiliations:** Department of Nutrition and Food Technology, Faculty of Agriculture, University of Jordan, Amman 11942, Jordan

## Abstract

There is a consensus among experts and consumers that pulses are a good source of nutrients and fiber. In a traditional hummus recipe, chickpeas are the major ingredient. The present study is aimed at developing new legume dips by exchanging chickpeas (Chd) with dry green (Gld) and red lentils (Rld), dry white beans (Wbd), and dry green peas (Gpd). Presoaking, boiling, proximate composition, pH, energy, color measurement, and sensory evaluation were conducted on the dips using chickpea dip (hummus) as a control. One-way ANOVA was used to determine the differences between the dips. The results revealed significant differences in the proximate composition of legume dips. The protein content of the five samples ranged between 7.46% and 9.19%, while the values varied from 8.59% to 10.93% in fat, 3.88% to 6.54% in crude fiber, 14.48% to 15.51% in carbohydrates, 171.95 to 195.13 in energy, 1.55% to 1.76% in ash, and 63.35% to 66.90% in moisture. These variations could be attributed to the type and composition of each legume, the soaking and boiling process, and the tahini added during the preparation. pH ranged between 4.5 and 4.7. The color measurement indicated that the five legume dips could be considered bright products (high L∗>67), with a positive color valuebluered-green and yellow-. Significant differences (*p* ≤ 0.05) were observed in the legume dips sensory evaluation, and the red lentil dip was the most acceptable with results comparable to the chickpea dip; it was followed by the green lentil, white bean, and green pea dips. These results highlight the feasibility of commercial production of legume dip that promotes human health and gives consumers more choices.

## 1. Introduction

It is anticipated that the world's population will reach 8.1 billion by 2025 and 9.6 billion by 2050. As the world's population continues to grow and as climate change threatens to disrupt the food supply, the prospect of widespread food shortages is becoming increasingly real. Both developed and developing countries are currently facing nutritional challenges that necessitate the implementation of effective solutions pertinent to alleviating protein energy malnutrition (in developing countries) and the imbalance of macro- to micronutrient consumption (in developed countries). Therefore, the agrifood industry has a great deal of opportunity and incentive to investigate alternative protein sources, such as plant-based proteins [1].

Being a member of the Leguminosae, legumes are an important source of inexpensive, good-quality protein for consumers. It has the most protein of any plant and meets about 10% of the total protein needs of people around the world [2]. Legumes comprise oilseeds, which include soybeans, peanuts, clover, and mesquite, and pulses, which consist of dry grains of peas, chickpeas, lentils, peas, beans, and lupins [3].

Among the various benefits they provide, consumption of pulses may lessen the risk of acquiring diet-related chronic illnesses (obesity, cardiovascular diseases, and type 2 diabetes), and is also associated with decreased total of blood cholesterol and low-density lipoprotein cholesterol (LDL-C). Furthermore, they have a nutrient profile consistent with weight control and antinutrients [4]. Moreover, the availability of legume-based products can help decrease meat consumption, which has proven effective in preventing diseases such as cancer and hypertension [5].

Legumes have nutritional, sensory, technological, and functional qualities. Similar to their nutritional values, they can be used as ingredients in many options. Many traditional foods in the Middle East are based on legumes of which fuol or medamis (stewed broad beans, Vicia faba, seasoned with garlic, lemon juice, and olive oil), falafel (deep fried flattened balls prepared from a mixture of previously soaked ground broad beans and chickpeas, garlic, onion, and a blend of herbs), and hummus are the most popular [6].

Hummus, chickpea dip, is a typical Mediterranean Arabic dish that has been popularized worldwide with the globalization of the food market. It is widely consumed by the entire population in Jordan, Syria, Lebanon, and other Arabic countries as a main breakfast dish, an appetizer snack, or in sandwich preparations. It is prepared with boiled, mashed chickpeas, tahini, lemon juice, and salt and topped with olive oil and spices, although other varieties exist [6]. Hummus is a highly nutritious food with about 6% protein, 15.7% carbohydrates, 1.7% ash, and 4.8% fat, as well as a moisture content is 71.0% and high water activity (0.98) [7]. Generally, the main ingredient in the preparation of hummus is chickpea (Cicer arietinum L.), which belongs to the Leguminosae family. It is a crucial component of the diets of people who cannot afford animal proteins or are vegetarians by choice [3]. This is due to the amount of protein in legumes (17–37%) being about the same as that in red meat (22–31%) [8].

Tahini (or sesame seed), the second major component in the hummus recipe, is obtained from mechanically hulled, roasted, and ground sesame seeds (*Sesamum indicum L.*) [9]. Tahini is consumed in Middle Eastern areas as a salad dressing, a dip, or a main component of many ready-to-eat foods such as hummus, foul moudamas, and baba ghanoush [10]. It has a positive effect on human health due to having high protein (>25%), fat (>45%), ash (<3.5%), and moisture (<1.5%) [11], with a shelf life at room temperature of one year [10].

Chickpeas and other legumes are similar in terms of their components and nutritional values. This has raised the question of whether it is possible to develop a product similar to hummus by exchanging chickpeas with other legumes. Accordingly, this study is aimed at developing hummus-like dips from dry red and green lentils, dry white beans, and dry green peas. These legumes were selected due to their availability, affordability, and high level of consumption in the Middle East in general, and Jordan in particular. The newly developed products offer different alternatives for both customers and factories to choose from. Studying their physical and chemical properties can increase the nutritional values of consumers. Furthermore, sensory evaluation can allow us to determine the customers' readiness to accept the new products when using hummus as a standard.

## 2. Materials and Methods

### 2.1. Materials

**Figure 1:**
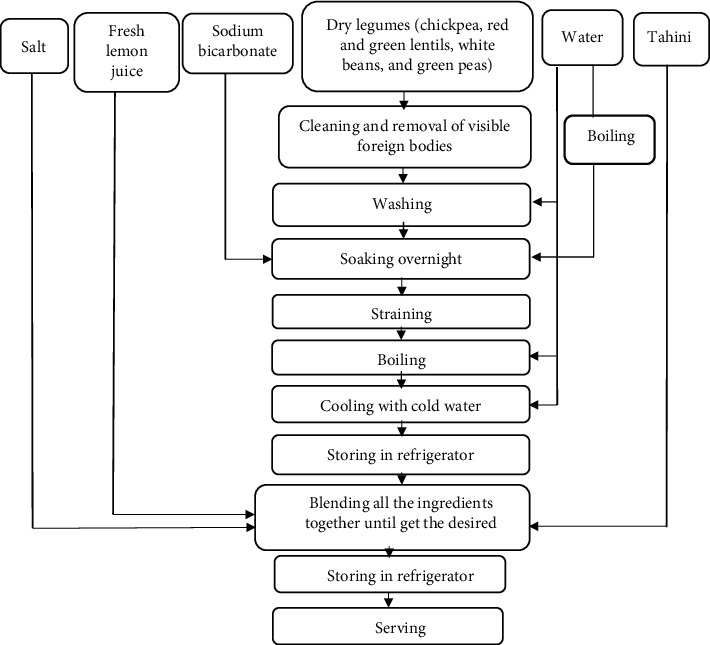
Preparation steps of legume dips [12].

Dry chickpeas, dry red and green lentils, dry white beans, and dry green peas, as well as the rest of the ingredients, namely, tahini, lemon juice, and salt, were collected from a local retail market in Amman, Jordan. The preparation of the dips was conducted under conventional hygienic conditions, following the recipe used by Yamani and Al-Dababseh [6] for hummus. It is worth noting that modifications have been made to the recipe to accommodate the legumes under study. The flow chart in Figure 1 demonstrates the steps taken in the preparation of the products [12].

### 2.2. Soaking and Boiling Methods

Five hundred grams of each dry legume was cleaned of foreign matter, washed, and then soaked overnight in boiled water (seed-to-water ratio of 1 : 3 (*w*/*v*)) at room temperature [13]. Sodium bicarbonate (1%) was added to the soaking water. After 12 hours of immersion, the soaking water was discarded, and the seeds were rinsed with tap water.

Taking the predetermined weight of soaked seeds, the legumes were put in tap water and cooked in a pot at 100 C at a water ratio of 1 : 4 (*w*/*v*) until soft [14]. Then, the surface of the treated seeds was dried using towel paper to eliminate the excess amount of water, and the weight of the samples was measured using a precision analytical balance (BTD-323, Phoenix Instrument, Blomberg, Germany). The water absorbed by the seeds was calculated according to Shafaei et al. [15].

The residue water boiling was used later in the step of product preparation. Because boiling water contains soluble proteins mobilized from the grains during cooking, it was decanted and saved for use in the preparation of products [16].

### 2.3. Preparation of the Legume Dips

One kilogram of each boiled legume was mashed with the other ingredients (detailed in Table 1) by using a conventional blender (Moulinex Fp247127) while gradually adding the water used in boiling until we got the desired texture. The ready products were packed in airtight plastic containers and labeled as follows: chickpea dip (Chd), red lentil dip (Rld), green lentil dip (Gld), white bean dip (Wbd), and green pea dip (Gpd).

### 2.4. Determination of pH

The AOAC method 981.12 was used to determine the pH of the samples [17]. The pH was measured by blending 10 g of the sample with 90 ml of distilled water, homogenized with a homogenizer for 30 seconds, and then measured using a calibrated pH meter (Hanna Instruments, Italy).

### 2.5. Proximate Composition

Moisture, protein, fat, ash, and crude fiber were determined according to the standard AOAC [18] official methods 925.09, 979.09, 920.39, 923.03, and 962.09, respectively. The total amount of carbohydrates was calculated by subtracting the sum of the other main constituents, including moisture, protein, ash, and fat from 100. To reduce errors, all tests were performed in triplicate.

### 2.6. Energy Determination

The total energy content of samples was computed using a conversion factor for each energy-yielding substrate of each food sample, where carbohydrate, protein, and fat yielded 4.0 kcal/g, 4.0 kcal/g, and 9.0 kcal/g of energy, respectively [18].

### 2.7. Color Measurement

The color of legume dips was measured using the colorimeter Hunter Lab Color Flex (Chroma Meter, CR-400, Konica Minolta, Sensing Inc., Japan). The determined color values were the mean of three readings taken for each sample and expressed in the color of L∗, a∗, and b∗, where L∗ indicates lightness from black (0) to white (100), a∗ describes the red-green color range with a∗>0 indicating redness and a∗<0 indicating greenness, and b∗ represents the yellow-blue color range with b∗>0 indicating yellowness and b∗<0 indicating blueness [19].

### 2.8. Sensory Evaluation

The sensory evaluation of the newly developed products was evaluated by 12 panellists selected from the Department of Nutrition and Food Technology at the University of Jordan. Among the panellists, 12 professors were taken as trained panellists to professionally assess each attribute of the five samples. The present study followed Lawless and Heymann's [20] ethical guidelines for an effective sensory evaluation test. Accordingly, the final products were placed in different dishes, which were carefully predisinfected and covered to ensure the safety of the products and to avoid any contamination. The panel was handed the food samples as well as a piece of bread and a bottle of water so that they could cleanse their palates between each tasting. An evaluation form was provided to each panellist, in which the food samples were coded with numbers to avoid the recognition of terms that might be influential. Panellists were asked to rate the products' overall acceptability, appearance, texture, smell, taste, and acidity on a nine-point hedonic scale, with 1 indicating extreme dislike and 9 indicating extreme liking.

### 2.9. Statistical Analysis

Statistical analysis of the data was carried out using the Statistical Analysis System Package (SAS Inc., 2000). A Tukey's test was performed to assess significantly different means. Moreover, an analysis of variance (ANOVA) was conducted, and a *t*-test was used to compare the means between sensory scores of treatments.

## 3. Results and Discussion

### 3.1. Soaking and Boiling Results

**Figure 2:**
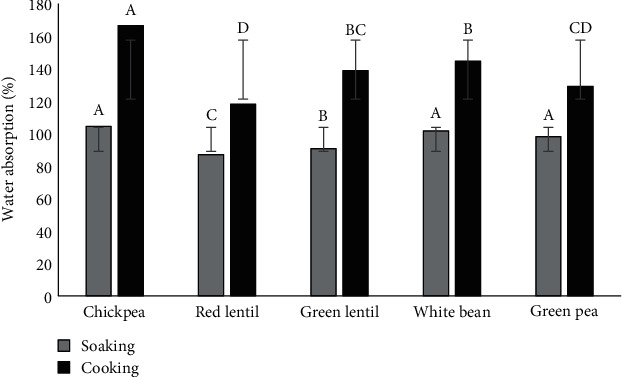
Water absorbed during the soaking and boiling of the legumes used in the preparation of the dips. Levels not connected by the same letter are significantly different (*p* ≤ 0.05) ± SD.

No significant differences were recorded between chickpeas, white beans, and green peas during soaking, and green and red lentils displayed significant differences. Chickpeas had the highest water absorption (104.40%) and red lentils (80.87%) had the lowest (see Figure 2). These results can be due to the size of each legume used in our study. Soaking causes the seeds to absorb water, which increases their size and weight [14]. Additionally, other factors may affect the water absorption of the legumes during soaking such as water temperature, soaking time, and some seeds' physical characteristics like hardness and seed coat thickness.

This process has certain advantages, such as reducing the antinutrient factors existing in dried legume seeds such as phytate, tannins, and oligosaccharides (raffinose, stachyose, verbascose, and ciceritol). These latter are also referred to as flatus-producing carbohydrates because they contain *α*-galactosidic bonds. The human body lacks the enzyme *α*-galactosidase, which is necessary to break these bonds. Hence, *α*-galactosidic is regarded as an antinutritional factor [21]. To reduce the oligosaccharide content, several methods are used, including soaking and boiling [22]. Han and Baik [23] found that soaking lentils, chickpeas, and green peas in tap water for 12 h reduced oligosaccharide content by 28%, 74.6%, and 56.3%, respectively. Furthermore, Vidal-Valverde et al. [24] reported that the total sugar content in chickpeas and kidney beans was decreased by 32% and 42% when boiled in water.

Except for lentils, the addition of sodium bicarbonate enhanced the softening of the chickpeas, white beans, and green peas. Sodium bicarbonate is usually added to soaking water to shorten the cooking time, because it breaks the pectate calcium and magnesium connections that are present in the tegument of beans, allowing for easier water absorption [25].

Boiling is the second step after soaking, which is the period from the commencement of boiling until 90-100% of the seeds are cooked, as measured by the standard method of determining the softness of the seeds by finger pressure [26]. In our study, the recorded boiling time for the samples ranged between 10 min for red lentils, 35 min for green lentils, 75 min for white beans, 90 min for green peas, and 105 min for chickpeas. The results obtained from the boiling of the five samples (see Figure 2) demonstrate significant differences between the weight variations. The absorbed water ranged from 119.60% (chickpeas) to 168.53% (red lentils). The weight of legumes was higher than the average values previously reported for dry legumes [27]. Thus, the factors of longer cooking time and higher temperature increase the rate of water uptake [28]. With regard to the cooking time, peas usually take up to 20 minutes [27]. However, the green peas used in the present study took longer than usual (90 min). This might be attributed to the variety of peas that were found in the area of Amman, Jordan. Furthermore, chickpeas and white beans are legumes that are “hard to cook” [28]. They took longer to cook than lentils.

### 3.2. pH Results

No significant differences were noticed in the pH of the legume dips, which were 4.55, 4.52, 4.53, 4.73, and 4.63 for chickpea dip, red lentil dip, green lentil dip, white bean dip, and green pea dip, respectively. Yamani and Al-Dababseh [6] reported a pH value of 5.1 in hummus, while Al-Qadiri et al., [29] recorded a value of 4.78, classifying it as a low-acid food. According to the Jordanian Standard N°465 [30] and CXS 257R [31], the total acidity of hummus should not exceed 1%, such as citric acid. As compared to the values reported in the literature, the samples in the present study have lower pH values. This decrease in pH could be due to the amount of lemon juice (which contains citric acid, which is antimicrobial) added during the preparation of dishes.

### 3.3. Proximate Composition

A significant difference was pertinent to the moisture content of the control sample chickpea dip and red lentil dip, green lentil dip, white bean dip, and green pea dip with the percentages of 66.70%, 63.35%, 65.35%, 66.01%, and 66.20%, respectively (Table 2). Aside from the red lentil dip, these results were in agreement with those found by Al-Qadiri et al. [29], who reported a 66.8% value of moisture content in hummus. As for the red lentil dip, moisture content was significantly lower than that reported by Takruri et al. [32], with a value of 65.01% in the chickpea dip. These variations could be attributed to the amount of water absorbed by the legumes during the soaking [33] and the amount of water added during the sample preparation to get the desired texture.

The protein content of the samples ranged between 7.46% and 9.19%, with red lentil dip having the highest protein content, followed by white bean dip, green pea dip, green lentil dip, and chickpea dip in that order (Table 2). These differences could be due to the soaking and cooking of the legumes, which causes a slight loss of nutrients like protein, minerals, and total sugar. It is noteworthy that the protein content of the legume dips is the sum of the legume protein and that of the next major dip ingredient, tahini [34, 35]. In the protein content of each legume, chickpeas, lentils, white beans, and green peas contain a high percentage of proteins, estimated at 24.41, 26.34, 22.48, and 22.25, respectively [36, 37], while tahini contains 23-27% protein [10]. In another study, Yamani and Isa [9] found that the percentage of protein in tahini was 24.7%.

Fat contents of 10.93%, 10.54%, 9.41%, 8.69%, and 8.59% for red lentil dip, green lentil dip, chickpea dip, white bean dip, and green pea dip, respectively, were significantly different. These results are in agreement with Wallace et al. [38], who found that the fat content of chickpea dip ranged from 4.83% to 12.3%. Pulses have no cholesterol and are often low in fat [39]. Hence, the main source of fat in these products is attributed to tahini, in which fat ranges from 57% to 65% [9, 40].

Significant differences in ash content *p* < 0.05 were noticed between samples (Table 2). Ash contents varied from 1.52% for red lentil dip to 1.76% for chickpea dip. Results were similar to those reported by Takruri et al. [41] whose findings revealed a value of 1.28%, and slightly lower than those of Al-Holy et al. [7] and Al-Qadiri et al. [29], who reported ash content results of 1.7% and 1.55-2%.

The carbohydrate content, as shown in Table 2, revealed no significant difference (*p* ≤ 0.05) between the samples. In particular, the values of chickpea dip, red lentil dip, green lentil dip, white bean dip, and green pea dip were 14.48%, 15%, 14.96%, 15.01%, and 15.51%, respectively. The results of the study were nearly similar to those obtained by Wallace et al. [38] and Amr and Yaseen [42], whose findings revealed a carbohydrate content of 14.29% and 15.7% in chickpea dip, respectively. Pulses have a total carbohydrate content (such as starch, polysaccharides, and other mono and oligosaccharides) ranging from 60 to 65%.

Dietary fiber is a component of plant-based diets that cannot be digested in the human small intestine [43]. Fibers are of two types, soluble (pectin and gums) and insoluble (cellulose, hemicellulose, and lignin). Soluble fiber can aid in weight management and cholesterol reduction, which is most advantageous for those who have diabetes and heart disease, while insoluble fiber assists with digestion [44]. It accelerates the transit of food through the stomach and intestines, increases bowel motility, promotes the growth of intestinal bacteria, improves gastrointestinal health, and lowers the risk of colorectal cancer by speeding up the elimination of waste from the digestive tract [45]. Noticeable differences (*p* < 0.05) in the crude fiber content of the five legume dips, the highest was in the white bean dip at 6.54%. This result is generally consistent with that found by Wallace et al. [38], who reported the value of crude fiber in hummus was 6%. Additionally, the findings were in agreement with those reported by Reister et al. [46] and Sokołowska et al. [47], in which the crude fiber content was 5.5% and 8.4%, respectively. While the crude values of the samples chickpea dip, red lentil dip, green lentil dip, and green pea dip were 5.66%, 4.94%, 4.28%, and 3.88%, in that order, these values were much higher than those obtained by Al-Holy et al. [7] and Takruri et al. [32], who reported that the crude fiber content in chickpea dip was 0.7% and 1.21%. These differences in crude fiber content could be attributed to the type of legumes used, as well as the soaking and boiling processes applied to them. Furthermore, pulses are the main source of dietary fiber in a hummus recipe. Tahini, with a fiber content of 1.8% [48], comes next to legumes as a main source of this component. Keyata et al. [49] found that the crude fiber in chickpeas was affected by different processing methods (direct grinding, dehulling, soaking, germinating, boiling, and dry roasting), where the average crude fiber content of the unprocessed chickpeas (5.81%) was significantly reduced to 5.16% after soaking and to 4.91% after boiling.

### 3.4. Energy Determination

The energy content of the samples ranged between 171.95 kcal and 195.13 kcal. The energy levels of red lentil dip (195.13 kcal) and green lentil dip (185.1 kcal) were significantly higher (*p* < 0.05) than those of white bean dip (173 kcal), chickpea dip (172.09 kcal), and green pea dip (171.95). These could be due to the high fat, carbohydrate, and protein content of legumes and tahini. According to Takruri et al. [32], the energy content of chickpea dip was 237 kcal. In another study by Reister et al. [46], they found that the value of energy in hummus commercials was 181 kcal.

### 3.5. Color Measurement

Color is one of the important quality attributes of foods that could influence their acceptability. There were significant differences between the five samples, where the L∗ values obtained for the legume dips ranged from 71.70 to 78.63 (Table 3). These values indicate that the legume dips studied are bright products (high L∗ value), while a∗ and b∗ values ranged from 2.68 to 6.35 and from 25.26 to 30.65, respectively. Ahmed et al. [50] reported that the L∗, a∗, and b∗ values of the hummus were 78.56, 2.77, and 27.01, respectively. In another study by Alvarez et al. [19], they found that the values of the colors L∗, a∗, and b∗ in hummus were 75.3, 0.29, and 14.3, respectively. The difference between the results of those studies and our results may be attributed to the color of each legume used, which contains several pigments at different levels, including carotenoids, chlorophyll, and flavonoids, as well as to tahini, whose values of L∗, a∗, and b∗ are 55.37, 5.61, and 12.23, respectively [51].

Color changes in products may be induced by several factors, including the action of such enzymes as polyphenol and other oxidase and chlorophyllases. Although enzymes in hummus are inactivated by heat treatment during the boiling of chickpeas and roasting of sesame seeds, some enzymatic activity may persist [52]. Güzel and Sayar outlined [53] the factors that can influence the color of foods during processing. The most prevalent are color degradation, browning reactions, and heavy metal contamination.

### 3.6. Sensory Evaluation

A sensory evaluation test was evaluated that provided five attributes, namely, appearance, smell, texture, taste, acidity, and overall acceptability of the prepared samples. This test was conducted to measure the sensory differences between legume dips and the control sample (chickpea dip) and to determine the consumers' opinions on newly developed products. The sensory evaluation scores of the new products indicate statistically significant differences between the five attributes and the overall acceptability (*p* ≤ 0.05) as reported by the study's panellists (Table 4).

The red lentil dip sample received the highest score in five of the attributes, with a score of 7.15 in taste, 7.73 in smell, 7.96 in texture, 7.01 in acidity, and a score of 7.63 in overall acceptability in comparison to chickpea dip, green lentil dip, white bean dip, and green pea dip. The reason why the red lentil dip sample was favored by panellists is generally due to its similarity to traditional chickpea dip in terms of appearance, taste, smell, and texture. It was followed by chickpea dip (the control sample), whose results were approximately similar to those of red lentils. Andersen et al. [5] reported that Danish food producers have tried a commercial version of hummus made from yellow peas as an alternative due to the similarity in taste, texture, and color between chickpea dip and yellow peas. Andersen et al.'s [5] study focused mainly on consumers' willingness to try and pay for hummus made from chickpeas, yellow peas, borlotti beans, carmencita beans, and lollandske rosiner produced in Denmark.

Although they could be considered generally acceptable dips, dips made from green lentils, white beans, and green peas received lower scores in terms of overall acceptability (6.97, 5.31, and 5.09, respectively) due to poor ratings in the categories of appearance, taste, and acidity. This can be attributed to the unusual appearance displayed by these newly developed products, which is significantly different from traditional hummus. The green lentil dip sample received a rating of “like slightly” on the hedonic scale, while the white bean dip and green pea dip samples received “neither like nor dislike.”

According to Hajas et al. [54], the addition of 15% of germinated green lentils affected the color, taste, and flavor of cookies. In their study, entitled “Consumers' opinions and choices using vegetable dip as an example product,” Mora et al. [55] examined the acceptability of vegetable dip, which consisted of beans, pumpkin, dried orange pulp and peel, lemon juice, olive oil, and tahini. The results of the sensory evaluation revealed a likeness to the newly developed product, which was further reinforced by the use of the term “fruits and vegetables.”

## 4. Conclusion

This study investigated the possibility of developing chickpea dip- (hummus-) like products from other legumes (dry green and red lentils, dry white beans, and dry green peas) that have high nutritional value and at the same time have sensory receptivity. Of all the legume dips studied, red legume dip was the best dip in terms of consumer preference and had the highest value of protein (9.19%). It is recommended that the product be marketed as an affordable, readily available alternative to the existing products on the market. The dips fit within a more realistic and accessible diet frame. Moreover, they can be interesting in populations following plant-based diets such as vegans and vegetarians as these dips are usually paired with bread, thus providing all the necessary amino acids. Further studies should be conducted to produce these dips on an industrial scale.

## Figures and Tables

**Table 1:** Ingredients of different legume dips, including chickpea dip (hummus) as control.

Basic ingredients (%)					
Dip	Mashed legume	Tahini	Fresh lemon juice	Salt	Boiling water
Chickpeas	48.60	14.58	12.76	0.73	23.33
Red lentils	62.34	18.70	11.78	0.94	6.24
Green lentils	56.69	17.01	10.71	0.85	14.74
White beans	56.67	17.00	11.31	0.85	14.17
Green peas	51.95	15.58	10.91	0.78	20.78

**Table 2:** Proximate composition of legume dips, including chickpea dip as control.

	Chd	Rld	Gld	Wbd	Gpd
Moisture	66.70^a^ ± 0.33	63.35^b^ ± 0.52	65.35^c^ ± 0.59	66.01^bc^ ± 0.27	66.20^ab^ ± 0.21
Protein	7.46^b^ ± 0.13	9.19^a^ ± 0.22	7.60^b^ ± 0.64	8.59^ab^ ± 0.28	8.15^ab^ ± 0.74
Fat	9.41^b^ ± 0.36	10.93^a^ ± 0.79	10.54^a^ ± 0.12	8.69^c^ ± 0.21	8.59^c^ ± 0.16
Ash	1.76^a^ ± 0.02	1.52^c^ ± 0.02	1.55^c^ ± 0.01	1.71^b^ ± 0.01	1.55^c^ ± 0.01
Carbohydrate	14.48^a^ ± 0.15	15.00^a^ ± 0.34	14.96^a^ ± 0.45	15.01^a^ ± 0.35	15.51^a^ ± 0.79
Fiber	5.66^b^ ± 0.30	4.94^c^ ± 0.91	4.28^d^ ± 0.46	6.54^b^ ± 0.36	3.88^d^ ± 0.08

Data are expressed as means of triplicate determinations. Levels not connected by the same letter are significantly different (*p* ≤ 0.05) ± SD. Chd: chickpea dip; Rld: red lentil dip; Gld: green lentil dip; Wbd: white bean dip; Gpd: green pea dip.

**Table 3:** Color of legume dips, including chickpea dip (hummus) as control.

Dip	L∗	a∗	b∗
Chd	74.47^b^ ± 0.79	5.57^a^ ± 0.27	30.66^a^ ± 0.83
Rld	71.70^c^ ± 0.99	6.35^a^ ± 0.31	30.65^a^ ± 0.18
Gld	67.86^d^ ± 0.11	3.43^bc^ ± 0.39	25.26^c^ ± 0.17
Wbd	78.63^a^ ± 0.60	4.05^b^ ± 0.45	29.63^a^ ± 0.46
Gpd	70.45^c^ ± 0.99	2.68^c^ ± 0.33	27.81^b^ ± 0.12

Data are expressed as means of triplicate determinations. Levels not connected by the same letter are significantly different (*p* ≤ 0.05) ± SD. L∗: lightness; a∗: red-green color; b∗: yellow-blue color; Chd: chickpea dip; Rld: red lentil dip; Gld: green lentil dip; Wbd: white bean dip; Gpd: green pea dip.

**Table 4:** Sensory evaluation of legume dips, including chickpea (hummus) as control, representing means for *n* = 12, where score 1 refers to dislike extremely and 9 to like extremely in the nine-point hedonic scale.

	Overall acceptability	Appearance	Smell	Texture	Teste	Acidity
Chd	7.45^ab^ ± 1.08	7.83^a^ ± 1.11	7.08^ab^ ± 1.56	7.66^ab^ ± 1.49	7.00^a^ ± 1.95	6.83^a^ ± 1.58
Rld	7.63^a^ ± 1.23	7.58^a^ ± 1.37	7.73^a^ ± 1.21	7.96^a^ ± 0.90	7.15^a^ ± 1.46	7.01^a^ ± 1.75
Gld	6.97^abc^ ± 1.83	4.91^b^ ± 1.72	6.83^ab^ ± 1.74	6.00^b^ ± 1.70	6.91^a^ ± 1.44	6.33^a^ ± 1.77
Wbd	5.31^bc^ ± 2.57	7.08^a^ ± 1.78	5.33^b^ ± 2.26	6.16^ab^ ± 1.99	6.00^a^ ± 2.08	5.66^a^ ± 2.26
Gpd	5.09^c^ ± 2.50	6.16^ab^ ± 1.58	6.25^ab^ ± 1.86	6.75^ab^ ± 1.65	5.25^a^ ± 2.45	6.16^a^ ± 2.03

Levels not connected by the same letter are significantly different (*p* ≤ 0.05) ± SD. Chd: chickpea dip; Rld: red lentil dip; Gld: green lentil dip; Wbd: white bean dip; Gpd: green pea dip.

## Data Availability

The data used to support the findings of this study are included within the article.
